# Fibroblasts: A Molecular and Pathophysiological Perspective

**DOI:** 10.3390/biomedicines13112754

**Published:** 2025-11-11

**Authors:** Dawid Wnuk, Milena Paw

**Affiliations:** Department of Cell Biology, Faculty of Biochemistry, Biophysics and Biotechnology, Jagiellonian University, Gronostajowa 7, 30-387 Kraków, Poland

## 1. Introduction

Since the first description in 1858 by Rudolf Virchow, who defined fibroblasts as cells found in connective tissue, the process of understanding the nature of fibroblasts has continued to this day [1]. Once considered as a structural cells responsible for extracellular matrix (ECM) synthesis and secretion, fibroblasts are now recognized as highly dynamic and heterogeneous cells responsible for tissue homeostasis, mediating immune responses, and stimulating pathological processes such as fibrosis, chronic inflammation, and tumor progression [2]. A systematic search of the PubMed database identified a total of 9570 articles investigating the role of fibroblasts in organ fibrosis published between 2020 and 2025. Since 1990, there has been a marked increase in mortality from diseases associated with tissue and organ fibrosis, in which activated fibroblasts play a key role—in 2019, these conditions accounted for approximately 35.4% of global deaths [3]. Contemporary research based on the large-scale single-cell and spatial transcriptomic atlases indicates the existence of a “universal” type of fibroblast, which is a single progenitor phenotype for further differentiation into activated subtypes with distinct transcriptional profiles and functional specializations [1,4,5,6]. Recent advances from 2020–2025 have provided a comprehensive understanding of the intrinsic molecular features of fibroblasts that predispose them to phenotypic switching: mechanotransduction and the involvement of YAP/TAZ signaling [7], metabolic reprogramming after hypoxia [8,9], shifts in cellular metabolism based on the switch from oxidative phosphorylation to glycolysis that have an impact on ECM synthesis and cytoskeletal remodeling [10], and active participation in intercellular communication and controlling immune responses [11]. Advanced three-dimensional culture systems, such as spheroids and engineered substrates with adjustable stiffness, have facilitated more physiologically relevant modelling of fibroblast behavior [12,13]. A comprehensive understanding of fibroblast biology and physiology, as well as their mechanistic involvement in the initiation and progression of fibrosis-related pathological processes, is crucial for the design and development of next-generation targeted therapies. Such insight is indispensable for enabling strategies that aim not only to prevent or halt fibrotic remodeling but also to reverse established fibrosis and restore normal tissue function. This Special Issue reflects the broad spectrum of current advances in our understanding of various aspects of fibroblast biology and their functions in both physiological and pathological processes. It comprises seven original research articles and one review, each offering unique insights that are briefly outlined in the following sections. We aim to underscore their significance and encourage readers to explore these contributions in greater depth.

## 2. An Overview of Published Articles

The first article published in this Special Issue [14] demonstrates for the first time that adult human bronchial fibroblasts (HBF) derived from non-asthmatic donors respond robustly to direct-current electric fields (dcEFs) by migrating directionally and re-orienting their long axes perpendicular to field lines, and that this sensitivity depends on both canonical and non-canonical TGF-β signaling pathways. In contrast, HBFs from asthmatic patients displayed markedly impaired electrotactic responses under identical conditions. These findings advance our understanding of the mechanobiological mechanisms governing airway regeneration by demonstrating that fibroblasts, in addition to airway epithelial cells [15], possess the capacity to sense electrical cues, and that this electrosensitivity may be dysregulated under pathophysiological conditions.

The second article, by Dirand et al. [16], evaluates whether three-dimensional spheroid cultures of keloid fibroblasts provide a relevant in vitro model for fibrogenesis in keloid research. The authors found that, contrary to expectations, fibrogenic features of keloid fibroblasts were markedly downregulated in 3D spheroid culture compared to traditional 2D monolayers. These findings suggest that while spheroid models may replicate aspects of the tissue microenvironment [17], they may not reliably mimic the fibrogenic behavior of keloid fibroblasts in vivo, thereby urging caution in using spheroids as a sole model for fibrogenesis in keloid research.

In the third article [18], the authors investigate the role of BRD3 in synovial fibroblasts isolated from patients with rheumatoid arthritis (FLS) by silencing BRD3 and analyzing transcriptome changes under TNF stimulation. The authors demonstrate that BRD3 serves as an upstream regulator of inflammatory, stress, and autophagy-related pathways in FLS, highlighting its crucial role in driving fibroblast activation in RA. These findings position BRD3 as a potential therapeutic target in RA-linked synovial pathology and related fibrotic processes

The fourth article by Dovrolis et al. [19] maps the interactions between stromal (including fibroblasts) and myeloid cell populations in the ileal mucosa of patients with Crohn’s disease, using single-cell RNA sequencing and in vitro validation, identifying the chemokine CCL-2 derived from fibroblasts as a key driver of monocyte recruitment. These findings highlight fibroblast-derived CCL-2 as a critical mediator of stromal–myeloid cell cross-talk in ileal Crohn’s disease. This study, together with another [20], reveals that spatial regulation of inflammation by fibroblast subpopulations could be a promising therapeutic target for interrupting persistent inflammation and altered immune–stromal interactions in the gut.

The following study published by Kato et al. [21] demonstrated that continuous NaF exposure significantly upregulated both gene and protein expression of FGF-2 and TGF-β in human gingival fibroblasts via CaMKII and ERK signaling pathways. These findings suggest a potential role for NaF in enhancing periodontal tissue repair during extraction sockets by promotion of fibroblast activity and connective tissue regeneration. It also further supports the clinical relevance of NaF mouthwash beyond caries prevention and highlights its therapeutic potential in oral tissue remodeling.

The sixth study, by Nájera-Martínez et al. [22], investigates the effects of trihalomethanes (THMs) on human lung fibroblasts (MRC-5), demonstrating that these environmental contaminants promote fibroblast activation and pro-inflammatory signaling through reactive oxygen species (ROS)-mediated modulation of the NF-κB pathway. Among the compounds examined, BrCHCl_2_ exhibited the highest binding affinity to the NF-κB/p65 complex, leading to enhanced dissociation of the IκBα-NF-κB complex and upregulation of pro-inflammatory gene transcription. These findings underscore a mechanistic link between THM exposure and fibrotic or inflammatory lung pathologies driven by redox imbalance and aberrant NF-κB activation.

Trejo Vazquez et al. in the seventh paper [23] demonstrates that extracellular vesicles (EVs) derived from lung adenocarcinoma (LUAD) cells are capable of activating lung fibroblasts into cancer-associated fibroblasts (CAFs), leading to upregulation of ECM-related and EMT-associated genes. These findings reveal that LUAD-derived EVs could be a key factor promoting CAF-mediated extracellular matrix remodeling and potentially facilitating epithelial-to-mesenchymal transition in cancer cells. The identification of EV-specific activation pathways suggests novel therapeutic targets to disrupt tumor–stroma interactions and impede metastasis in lung cancer.

The last paper included in this Special Issue, from Somnay et al. [24], presents a comprehensive synthesis of liver fibrosis mechanisms, leading to the accumulation of extracellular matrix secreted by activated hepatic stellate cells, myofibroblasts, and inflammatory immune cells. Although advanced cirrhosis is traditionally considered irreversible, recent evidence suggests that early stages of fibrosis may be reversible, and emerging therapies targeting fibrogenic pathways offer new therapeutic potential.

## 3. Conclusions

This Special Issue presents a comprehensive collection of studies that collectively underscore the pivotal role of fibroblasts in regulating tissue homeostasis and driving pathological remodeling across diverse organ systems. The eight contributions consistently demonstrate that fibroblasts are not merely structural components but dynamic cellular players whose activation states critically influence disease initiation and progression. Despite the wide range of pathological contexts examined—spanning asthma, keloids, rheumatoid arthritis, Crohn’s disease, periodontal repair, environmental lung injury, cancer, and liver fibrosis—all studies converge on the central finding that fibroblast activation is a key determinant of disease outcomes.

A unifying theme throughout these investigations is the identification of signaling pathways and microenvironmental interactions as fundamental drivers of organ fibrosis and inflammatory dysregulation. These insights emphasize that fibroblast heterogeneity, including their intrinsic molecular diversity, is central to understanding how different fibroblast subsets contribute to disease-specific pathophysiology. The heterogeneity of fibroblast subpopulations and their distinct roles within complex cellular and microenvironmental interactions are only now being uncovered through advances in methodologies such as single-cell RNA sequencing, proteomics, and three-dimensional in vitro models. These technologies also facilitate the identification of new molecular targets for therapeutic intervention.

Although intensive research and literature data reveal significant gaps in knowledge concerning the mechanisms governing fibroblast activation, particularly in relation to their interactions with immune cells and the extracellular matrix. The contributions collectively highlight the need for designing advanced and physiologically relevant research models that accurately reflect the dynamic interactions between fibroblasts, immune cells, and the surrounding microenvironment. They also indicate that targeting fibroblast activation pathways is a promising strategy for the development of precision therapeutics aimed at preventing or limiting organ fibrosis [25]. Importantly, the identification of shared fibroblast activation mechanisms across tissues suggests that cross-organ antifibrotic therapies may have an important clinical relevance [26]. Collectively, the contributions provide a unified framework for understanding fibroblast-driven mechanisms across diseases and lay the groundwork for future fibroblast-targeted therapeutic strategies.
